# Prognostic Value of Radiological Extranodal Extension Detected by Computed Tomography for Predicting Outcomes in Patients With Locally Advanced Head and Neck Squamous Cell Cancer Treated With Radical Concurrent Chemoradiotherapy

**DOI:** 10.3389/fonc.2022.814895

**Published:** 2022-05-27

**Authors:** Abhishek Mahajan, Ankur Chand, Ujjwal Agarwal, Vijay Patil, Richa Vaish, Vanita Noronha, Amit Joshi, Akhil Kapoor, Nilesh Sable, Ankita Ahuja, Shreya Shukla, Nandini Menon, Jai Prakash Agarwal, Sarbani Ghosh Laskar, Anil D’ Cruz, Pankaj Chaturvedi, Devendra Chaukar, P. S. Pai, Gouri Pantvaidya, Shivakumar Thiagarajan, Swapnil Rane, Kumar Prabhash

**Affiliations:** ^1^ Department of Radiodiagnosis and Imaging, Tata Memorial Hospital, Homi Bhabha National Institute, Mumbai, India; ^2^ Department of Medical Oncology, Tata Memorial Hospital, Homi Bhabha National Institute, Mumbai, India; ^3^ Department of Head and Neck Surgical Oncology, Tata Memorial Hospital, Homi Bhabha National Institute, Mumbai, India; ^4^ Department of Radiation Oncology, Tata Memorial Hospital, Homi Bhabha National Institute, Mumbai, India; ^5^ Department of Pathology, Tata Memorial Hospital, Homi Bhabha National Institute, Mumbai, India

**Keywords:** diagnostic imaging, computed tomography, extranodal extension, survival, neoplasm staging, radiology, oral cancers, squamous cell carcinoma

## Abstract

**Objective:**

Extra Nodal Extension (ENE) assessment in locally advanced head and neck cancers (LAHNCC) treated with concurrent chemo radiotherapy (CCRT) is challenging and hence the American Joint Committee on Cancer (AJCC) N staging. We hypothesized that radiology-based ENE (rENE) may directly impact outcomes in LAHNSCC treated with radical CCRT.

**Materials and Methods:**

Open-label, investigator-initiated, randomized controlled trial (RCT) (2012–2018), which included LAHNSCC planned for CCRT. Patients were randomized 1:1 to radical radiotherapy (66–70 grays) with concurrent weekly cisplatin (30 mg/m^2^) [cisplatin radiation arm (CRT)] or same schedule of CRT with weekly nimotuzumab (200 mg) [nimotuzumab plus CRT (NCRT)]. A total of 536 patients were accrued and 182 were excluded due to the non-availability of Digital Imaging and Communications in Medicine (DICOM) computed tomography (CT) data. A total of 354 patients were analyzed for rENE. Metastatic nodes were evaluated based on five criteria and further classified as rENE as positive/negative based on three-criteria capsule irregularity with fat stranding, fat invasion, and muscle/vessel invasion. We evaluated the association of rENE and disease-free survival (DFS), loco-regional recurrence-free survival (LRRFS), and overall survival (OS).

**Results:**

A total of 244 (68.9%) patients had radiologically metastatic nodes (rN), out of which 140 (57.3%) had rENE. Distribution of rENE was balanced in the two study groups CRT or NCRT (p-value 0.412). The median follow-up period was 39 months (ranging from 35.5 to 42.8 months). Complete response (CR) was seen in 204 (57.6%); incomplete response (IR), i.e., partial response plus stable disease (PR + SD), in 126 (35.6%); and progressive disease (PD) in 24 (6.8%). rENE-positive group had poor survival compared to rENE-negative group 3-year OS (46.7% vs. 63.6%), poor DFS (48.8% vs. 87%), and LRRFS (39.9% vs. 60.4%). rENE positive had 1.71 times increased risk of IR than rENE negative. Overall stage, site, clinical metastatic node (cN), response, and rENE were the significant factors for predicting OS, DFS, and LRRFS on univariate analysis. After making adjustment on multivariate analysis, rENE was an independent prognostic factor for DFS and trending to be significant for OS.

**Conclusion:**

Pre-treatment rENE is an independent prognostic marker for survival in patients with LAHNSCC treated radically with CCRT that can be used as a potential predictive marker for response to treatment and hence stratify patients into responders vs. non-responders. We propose the mahajan rENE grading system applicable on CT, magnetic resonance imaging, positron emission tomography–contrast-enhanced CT, and ultrasound.

## Introduction

The commonest cancer histology in the head and neck is squamous cell carcinoma (SCC) (1). CCRT is a standard treatment option in LAHNSCC. It serves either as definitive treatment in cancers of oropharynx, larynx, and hypopharynx or as adjuvant treatment for oral cavity cancers post-surgery in presence of pathological ENE (pENE) or positive tumor margins on histopathology (2, 3).

ENE is an important prognostic and predictive factor that has been accorded a place in American Joint Committee on Cancer (AJCC) eight edition N category for head and neck cancers and has become the criteria for N3b disease (4). pENE has been extensively studied, and it can be microscopic (ENEmi) or macroscopic (ENEma). A study by Tirelli et al. found that the 3-year OS was 46% in the ENEmi group and 38.9% in the ENEma group (5). Another study by Thomas et al. found that pENE and clinical ENE (cENE) were associated with 60% decrease in 5-year OS compared to negative ENE (6). However, as ENE is pathologically assessed, this important information is not available for patients who are radically treated with definitive chemoradiotherapy and histopathological ENE is not available. Hence, imaging-based assessment of ENE [radiology-based ENE (rENE)] using various imaging criteria can help in better prognostication and planning with the intensification of adjuvant treatment in LAHNSCC. Few recent studies have shown the clinical implications of rENE in head and neck cancers (7). A study by Benjamin et al. found that rENE-positive patients had significantly worse 3-year OS (95% vs. 77%), progression-free survival (91% vs. 71%), and distant control (98% vs. 81%) than rENE-negative patients in locally advanced oropharyngeal carcinoma (8).

Multiple imaging-based studies have been performed in the past to know the diagnostic accuracy of rENE using CT, magnetic resonance imaging (MRI), and ultrasonography (US) and correlating it with pENE. Few studies have compared the diagnostic accuracy of different imaging modalities; for example, the meta-analysis by Su et al. (9) reported comparable results between CT and MRI in predicting ENE with sensitivity and specificity of 77% and 85% for CT and 85% and 84% for MRI, respectively. They also found sensitivity of 86% and specificity of 86% for positron emission tomography–computed tomography (PET-CT) and of 87% and 75%, respectively, for ultrasound (US). Almulla et al. showed that compared to MRI, CT showed improved sensitivity, negative predictive value, and diagnostic accuracy but similar specificity and positive predictive value (10). CT has been found reliable for evaluating rENE, with sensitivity ranging from 64% to 73% and specificity ranging from 82% to 87%, respectively. A study by Url et al. found sensitivity of CT in rENE detection of 73% and specificity of 91% (11). Given the high specificity and negative predictive value, it is worthwhile to explore implications of rENE in patients treated radically with CCRT, where pENE is not available. CT-based rENE has the potential to be used to accurately stage by predicting ENE and to stratify patients into high and low risk before planning the radical treatment in LAHNSCC.

With this background, we hypothesize that rENE and hence the N category based on it may have a potential to be prognostic and predictive marker in determining outcomes, we performed this analysis on the dataset available from already published randomized controlled trial (CTRI/2014/09/004980) (2). The present study is a *post hoc* analysis that evaluates the impact of rENE on survival outcome on a cohort of patients who were prospectively followed up.

## Materials and Methods

### Materials

**Figure 1:**
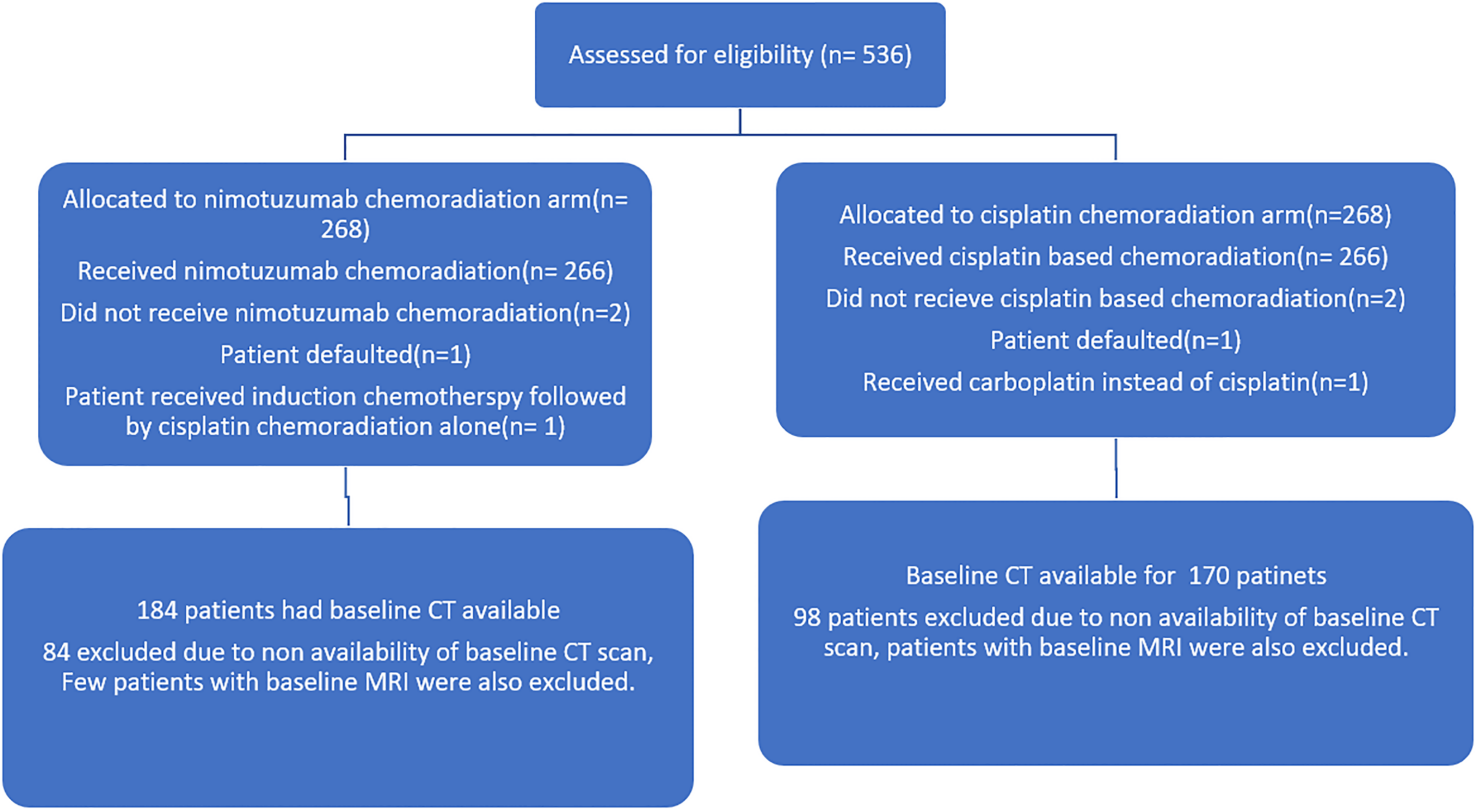
Consort diagram of the nimotuzumab plus cisplatin-chemoradiation arm versus cisplatin-chemoradiation arm showing the patient selection.

The study was approved by institutional ethics committee and was registered with clinical trial registry of India (CTRI/2014/09/004980). All patients underwent standard study protocol for evaluation and treatment. This was an investigator-initiated, randomized controlled trial, which allocated LAHNSCC (AJCC seventh edition stage III and IV oropharyngeal, laryngeal, and hypopharyngeal cancer) patients in ratio of 1:1 to receive either radical radiotherapy (66–70 Gy) with concurrent weekly cisplatin (30 mg/m^2^) (CRT) or the same schedule of CRT with weekly nimotuzumab (200 mg) (NCRT). The stratified block randomization was performed and trial population was stratified by five factors, namely, site of malignancy (oropharynx versus others), stage (III versus IV), age (≤60 versus >60 years), radiotherapy technique, and treatment center. The key inclusion criteria for the current analysis were treatment-naive oropharyngeal, laryngeal, and hypopharyngeal patients with pre-treatment contrast-enhanced CT (CECT)/PET-CT available in our institute. Patients who had received any treatment prior, unavailable DICOM data on imaging or who presented with distant metastasis, inadequate treatment details, and follow-up data were excluded. Detailed inclusion and exclusion criteria of the trial are mentioned in the supplement of the main published article of the trial (2). A total 536 patients were enrolled in this trial between 2012 and 2018. Out of these, 182 patients were excluded from this analysis due to the exclusion criteria mentioned above. The final analysis was performed on a cohort of 354 patients: 181 oro-pharyngeal and 173 other sites (larynx and hypopharynx) cancers. The consort diagram is shown in **Figure 1**.

#### Treatment Protocol and Follow-Up

Both the arms were administered high-dose, curative radiotherapy for 6.5 to 7 weeks using a standard two-dimensional (2D) technique, a three-dimensional (3D) conformal technique, or intensity-modulated radiotherapy with megavoltage radiation. Local tumor and lymph node disease were treated with 70 grays (Gy), in 2 Gy per fraction, 5 days per week, whereas upto dose of 46 to 50 Gy was planned for uninvolved nodal regions of the neck. Other altered fractionation schedules were permitted if the biologic equivalent dose for tumor control was similar to 70 Gy at 2 Gy per fraction. Cisplatin was given at a dose of 30 mg/m^2^ weekly during radiation along with supportive medication in both arms. Weekly Nimotuzumab in addition was given in the NCRT arm intravenously as a 200-mg dose in 250 ml of normal saline over 60 minutes without any premedication. The response assessment of PET-CECT was performed 8 weeks after the completion of active treatment. The baseline imaging was performed prior to the initiation of treatment and subsequent imaging performed as per the schedule, which was compared for Response Evaluation Criteria in Solid Tumours (RECIST). The patients were followed up clinically and with CECT at 12 and then 24 weeks from randomization for initial 6 months. Subsequently, patients underwent clinicoradiological evaluation every 6 months for the next 2 years. After the completion of 2 years, patients were followed up only clinically until the completion of 5 years. Regional recurrences that were detected on clinical examination or imaging were confirmed with fine needle aspiration cytology.

### Image Analysis

**Figure 2:**
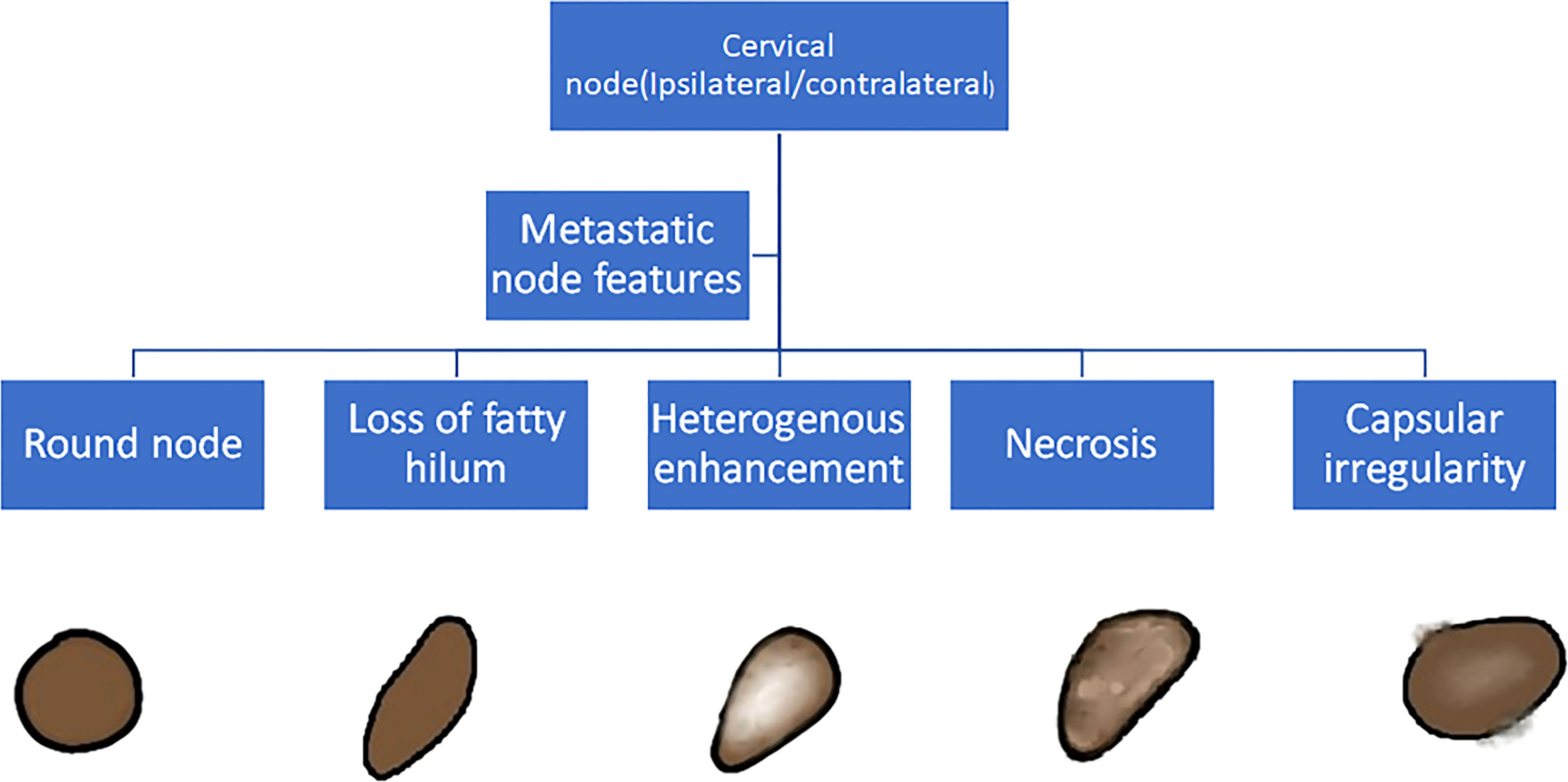
Criteria to classify a node as metastatic: Round node, loss of fatty hilum, heterogeneous enhancement, necrosis, and capsular irregularity. Node with any 2 or more positive features was considered metastatic.

**Figure 3:**
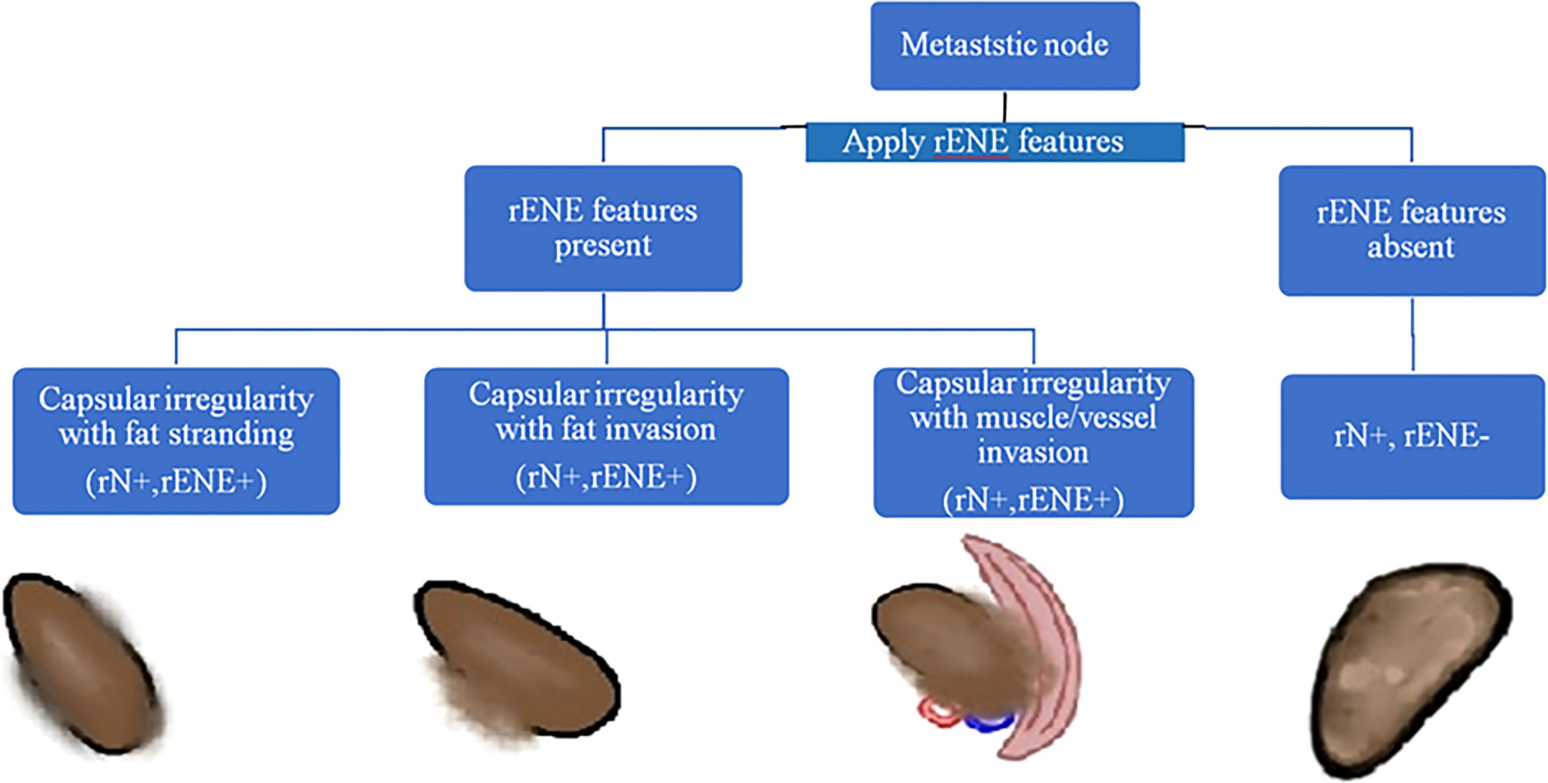
Algorithm to determine ENE positive/negative status. Node with the presence of any one of the three features, namely, capsular irregularity with fat stranding, capsular irregularity with fat invasion, or capsular irregularity with gross muscle/vessel invasion, was considered ENE positive.

The baseline imaging data (CECT images) of the eligible patients were reviewed by two dedicated head and neck radiologists (AM and NS with 10 and 7 years of experience, respectively) independently. Nodes were categorized into metastatic and non-metastatic and further into rENE positive and rENE negative. The criteria used for identifying metastatic node included round shape, loss of fatty hilum, necrosis, heterogeneous enhancement, and irregular capsule (**Figure 2**). Nodes with the presence of any two or more criteria out of these five criteria were considered positive for nodal metastasis to improve the specificity. The criteria for rENE included capsular irregularity with fat stranding, capsule irregularity with fat invasion and gross muscle/vessel invasion (**Figure 3**). rENE was considered positive if any one criterion was present. The final metastatic and rENE was reported patientwise, i.e., patients were defined as node and rENE positive and negative. The individual features were reported independently by both the radiologist, and if there was discordance between the final status of nodal metastasis or rENE, then both the radiologists reviewed the scans together and reached a consensus. We evaluated the association of rENE and clinical outcomes.

### Statistical Analysis

The patients’ demographics, treatment, and outcome data were entered in IBM SPSS version 21.0. The Chi-square test and Fisher’s exact test were used to analyze descriptive data. The value of p < 0.05 was considered statistically significant. Kaplan–Meier estimates were performed for survival analysis, and comparison was done using log-rank test. The OS was calculated from the date of randomization to the date of death. DFS was calculated from the time when the patient was disease-free, i.e., if the patient was disease-free (achieved complete remission) after chemoradiotherapy, then it was calculated from the date of response assessment and in patients of residual disease that underwent salvage surgery; it was calculated from the date of salvage surgery to the date of any locoregional or distant disease recurrence. In all other patients who were never disease-free, it was considered 0. LRRFS was calculated from the date of randomization to the date of local and/or regional recurrence. Univariate cox analysis was performed to calculate hazard ratio for potential prognostic factors, including age at diagnosis, gender, site of tumor, stage of tumor (cT category, cN category, and overall stage), response, rENE, cumulative cisplatin dose >200 mg/m^2^, and treatment regime. Statistically significant factors on univariate analysis and clinically important covariates were used to create a multivariate Cox proportional hazards regression model to determine whether rENE is an independent prognostic factor.

## Results

### Patient Characteristics

The median follow-up period was 39 (ranging from 35.5 to 42.8) months. Out of 354 patients, 302 (85%) were male and 52 (15%) were females (summarized in **Table 1**). The median age at diagnosis was 54 years (range, 20–77 years). A total of 181 (51.1%) patients had oropharynx, 98 (27.7%) patients had larynx, and 75 (21.2%) had hypopharynx cancer. Most of the tumors were poorly differentiated (30.4%). Out of 354 patients, 158 (44.7%) had a recurrence. Out of which, local recurrence occurred in 58 (36.7%), nodal recurrence in 35 (22.2%), distant recurrence in 33 (20.9%) patients, and 32 (20.3%) patients showed combination. There were 146 (41.2%) deaths during the follow-up, out of which 117 (80.1%) were due to disease and 29 (19.8%) were due to other causes.

**Table 1:** Patient characteristics.

Clinical variables	Number (n = 354)	Percentage (%)
Gender (Male/female)	302/52	85/15
Age (years, median)	54 (20–77)	
Site		
Oropharynx	181	51.1
Hypopharynx	75	21.2
Larynx	98	27.7
T stage		
T1, T2	89	25.1
T3, T4	265	74.9
Clinical node(cN)		
cN+	264	74.6
cN−	90	25.4
Radiological positive node(rN)		
rN+	244	69
rN−	110	31
Radiological extranodal extension(rENE)		
rENE+	140/244	57.4
rENE−	104/244	42.6
Response		
CR	204	57.6
PD	24	6.8
IR (SD + PR)	126	35.6
Status		
Alive	208	58.8
Deaths	146	41.2
Deaths		
Death due to disease	117/146	80.1
Death due to other cause (drug toxicity, second primary, tuberculosis, and unknown)	29/146	19.9
Histopathological differentiation		
WDSCC	2	0.6
MDSCC	65	18.3
PDSCC	107	30.2
SCC not specified	180	50.9
Recurrence		
Present	158	44.7
Absent	196	55.3
Recurrence site		
Local	58	36.7
Nodal	35	22.2
Distant	33	20.9
Combination	32	20.3

CR, complete response; PR, partial response; PD, progression of disease; SD, stable disease; IR, incomplete response; WDSCC, well-differentiated squamous cell carcinoma; MDSCC, moderately differentiated squamous cell carcinoma; PDSCC, poorly differentiated squamous cell carcinoma; SCC, squamous cell carcinoma.

### Imaging Characteristics

**Figure 4:**
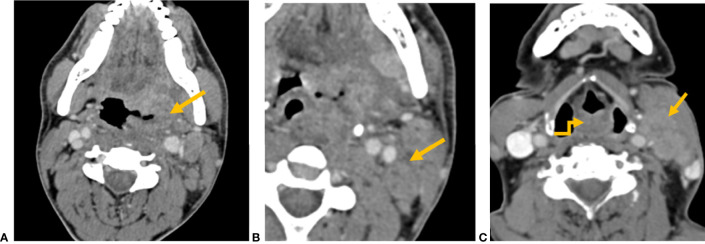
CT axial image shows **(A)** left tonsillar fossa SCC (yellow arrow) and **(B)** metastatic left level II node showing capsular irregularity and surrounding fat stranding (yellow arrow), representing extranodal extension. **(C)** CT axial image shows an ill-defined mass involving the aryepiglottic fold (yellow elbow arrow). Metastatic left level II node with gross muscle invasion represents extranodal extension (yellow arrow).

There were 264 (74.6%) patients with clinically positive nodes (cN). A total of 244 had radiological positive nodes (rN), out of which 140 (57.4%) were ENE positive. The number of nodes that were clinically reported as metastatic were significantly more than radiologically metastatic nodes (p < 0.001). Among 244 nodes that were radiologically metastatic, 231 (94.7%) nodes showed round shape, 230 (94.3%) with loss of fatty hilum, 210 (86.1%) showed heterogeneous enhancement, 196 (80.3%) with capsular irregularity, and 172 (70.5%) showed necrosis. Among 140 rENE-positive patients, capsular irregularity with fat stranding was present in 140 patients (100%), capsular irregularity with fat invasion in 64 (45.7%) patients, and 53 (37.9%) had muscle/vessel invasion. Necrosis was present in 128 (91.4%) rENE-positive patients. Imaging characteristics have been summarized in **Table 2** and their examples in **Figure 4**.

**Table 2:** Imaging characteristics.

rN (n = 244)		rENE (n = 140)	
Features	Number (n)/Percentage	Features	Number (n)/Percentage
Round node	231/94.7	Capsular irregularity with fat stranding	140/100
Loss of fatty hilum	230/94.3	Capsular irregularity with fat invasion	64/45.7
Heterogenous enhancement	210/86.1	Capsular irregularity with muscle/vessel invasion	53/37.9
Capsular irregularity	196/80.3		
Necrosis	172/70.5		

#### CRT Versus NCRT Arm

The baseline CT imaging was available in 170 patients in the cisplatin chemoradiation arm and 184 patients in nimotuzumab chemoradiation arm. The presence of rENE was distribution was balanced in both the treatment arms, i.e., 71 (41.8%) rENE-positive patients in CRT arm vs. 69 (37.5%) rENE-negative patients in NCRT arm (p = 0.41). The cumulative dose of cisplatin 200 mg/m^2^ was given equally in rENE-positive 117 (83.6%) and rENE-negative patients 171 (79.9%) (p = 0.39).

#### OS, DFS, and LRRFS Univariate Analysis

**Figure 5:**
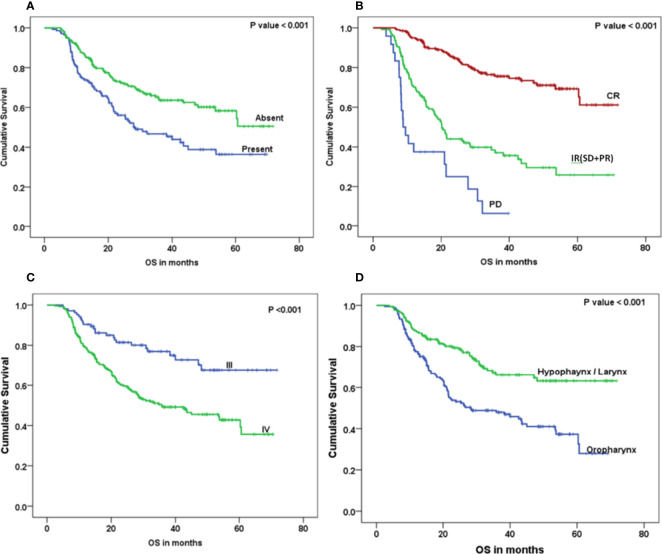
Kaplan–Meier survival curves showing overall survival (OS) by clinical factors: **(A)** rENE, **(B)** response, **(C)** overall stage, and **(D)** site.

**Figure 6:**
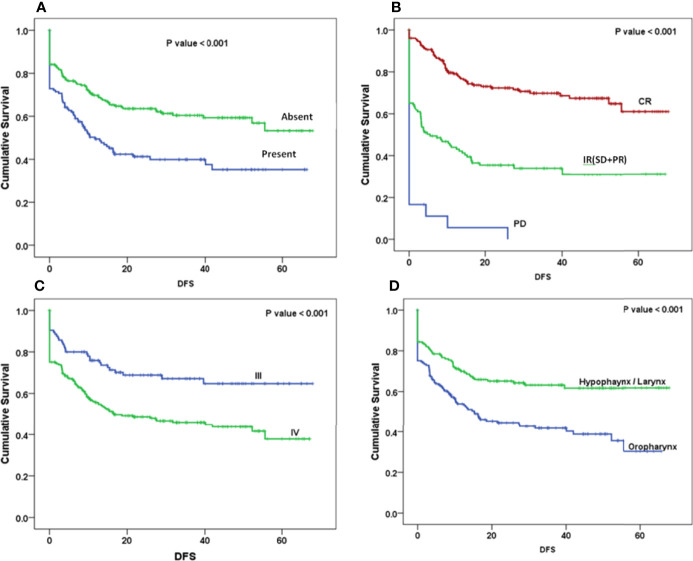
Kaplan–Meier survival curves showing disease-free survival (DFS) by clinical factors: **(A)** rENE, **(B)** response, **(C)**, overall stage, and **(D)** site.

**Figure 7:**
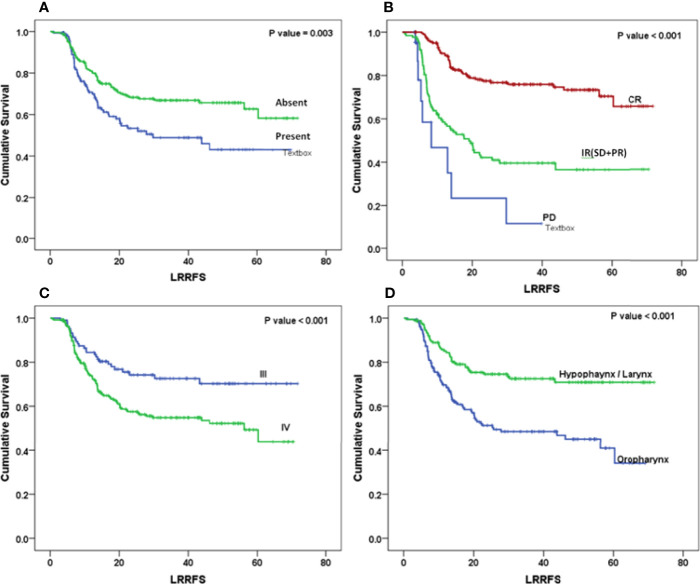
Kaplan–Meier survival curves showing locoregional recurrence-free (LRRFS) survival by clinical factors: **(A)** rENE, **(B)** response, **(C)** overall stage, and **(D)** site.

The Kaplan–Meier curve (**Figures 5**–**7**) showed 3-year OS, DFS, and LRRFS for the hypopharynx–larynx were significantly higher as compared to oropharynx (p < 0.001). Patients with the clinically node positive disease had poor survival than the clinically node negative disease (p = 0.001). Stage IV had poor survival than stage III patients (p < 0.001). When the response was assessed according to RECIST criteria, CR had better survival than PR, and PD showed worst survival (p < 0.001). When compared to rENE status, patients with rENE-positive status had poor survival [OS (p < 0.001), DFS (p < 0.001), and LRRFS (p = 0.003)] compared to rENE-negative patients (**Figures 5**–**7**). On performing the cox univariate analysis gender, site, cN, stage, rENE, and response had a significant impact on OS (**Table 3**). Site, cN, stage, rENE, response, and treatment arm were significant prognostic factors for DFS and LRRFS (**Table 3**).

**Table 3:** Univariate analysis for OS, DFS and LRRFS.

Variables		n	HR 95 % (CI)	P-value	HR 95 % (CI)	P-value	HR 95 % (CI)	P-value
			OS		DFS		LRRFS.	
Age	<60	256	1.266 (0.869–1.845)	0.218	1.203 (0.844–1.715)	.283	1.346 (0.892–2.029)	0.155
	≥60	98	1		1		1	
Gender	Male	302	1.794 (1.033–3.113)	**0.035**	1.485 (0.909–2.424)	.096	1.758 (0.97–3.189)	0.060
	Female	52	1		1		1	
Site	Oropharynx	181	2.15 (1.529–3.023)	**<0.001**	1.856 (1.349–2.552)	**<0.001**	2.322 (1.6–3.369)	**<0.001**
	Hypopharynx- Larynx	173	1		1		1	
T stage	T1-T2	89	1		0.921 (0.653–1.299)	.622	0.969 (0.653–1.437)	0.876
	T3-T4	265	1.138 (0.783–1.653)	0.498	1		1	
Clinical node	cN+	264	2.09 (1.358–3.218)	**0.001**	2.098 (1.386–3.176)	**<0.001**	2.206 (1.378–3.531)	**0.001**
	cN−	90	1		1		1	
Overall Stage	IV	249	2.396 (1.557–3.687)	**<0.001**	1.949 (1.329–2.859)	**.001**	1.901 (1.241–2.913)	**0.003**
	III	105	1		1		1	
rENE	Present	140	1.799 (1.3–2.491)	**<0.001**	1.819 (1.335–2.479)	**<0.001**	1.707 (1.199–2.429)	**0.003**
	Absent	214	1		1		1	
Response	PD	24	9.344 (5.483–15.925)	**<0.001**	8.925 (5.353–14.882)	**<0.001**	7.535 (3.664–15.497)	**<0.001**
	IR (SD + PR)	126	3.932 (2.737–5.65)	**<0.001**	3.24 (2.306–4.553)	**<0.001**	3.5 (2.411–5.082)	**<0.001**
	CR	204	1		1		1	
Cisplatin 200 mg	No	66	1.103 (0.742–1.640)	0.629	1.047 (0.711–1.542)	.815	0.943 (0.6–1.483)	0.799
	Yes	288	1		1		1	
Treatment arm	CRT arm	170	1.160 (0.838–1.604)	0.370	1.362 (1–1.856)	**0.050**	1.472 (1.034–2.094)	**0.032**
	NCRT arm	184	1		1		1	

HR, hazard ratio; CI, confidence interval; IR, incomplete response.Bold values means statistically significant.

#### OS, DFS, and LRRFS Multivariate Analysis

On multivariate analysis, the independent prognostic factors for OS were site (oropharynx vs. laryngo-hypopharynx, p = 0.004) and stage (stage III vs. stage IV, p = 0.015). The presence of rENE had a trend toward inferior OS (p = 0.069) (**Table 4**). rENE was an independent prognostic factor of DFS (p = 0.021) along with the site of disease (p = 0.017) (**Table 4**). Only site and the treatment arm (CRT vs. NCRT) were independent prognostic factors for LRRFS (**Table 4**).

**Table 4:** Multivariate analysis for OS, DFS, and LRRFS.

Variables	P-value	HR	95.0% CI for Exp(B)		P-value	HR	95.0% CI for Exp(B)		P-value	HR	95.0% CI for Exp(B)	
			Lower	Upper			Lower	Upper			Lower	Upper
	OS				DFS				LRRFS			
CRT vs. NCRT arm	0.462	1.131	0.815	1.568	0.078	1.322	0.969	1.803	**0.045**	1.437	1.008	2.050
Age	0.216	1.269	0.870	1.853	0.359	1.182	0.827	1.691	0.171	1.336	.883	2.021
Site	**0.004**	1.713	1.191	2.464	**0.017**	1.518	1.079	2.136	**0.001**	1.990	1.333	2.971
Cisplatin 200 mg	0.368	1.204	0.804	1.805	0.475	1.155	0.778	1.714	0.625	1.122	0.707	1.781
Overall Stage	**0.015**	0.566	0.358	0.894	0.072	0.686	0.454	1.034	0.202	0.743	0.471	1.173
rENE	0.069	1.370	0.976	1.925	**0.021**	1.466	1.059	2.029	0.172	1.293	0.894	1.871

Bold values means statistically significant.

#### Factors Predicting Clinical Response

Site, cN, and rENE were significant factors for predicting the response to treatment both on univariate and multivariate analysis (**Tables 5**, **6**). rENE-positive patients had 1.71 times increased risk of IR than negative patients.

**Table 5:** Univariate analysis to determine the factors predicting complete response.

Variables		Patients with complete response (%)	P-value
Age	<60	143 (56)	0.277
	≥60	61 (62)	
Gender	Male	171 (57)	0.357
	Female	33 (64)	
Site	Oropharynx	87 (48)	**<0.001**
	Hypopharynx- Larynx	117 (67)	
T stage	T1-T2	53 (60)	0.671
	T3-T4	151 (57)	
Clinical node	cN−	69 (77)	**<0.001**
	cN+	135 (51)	
rENE	Present	62 (44)	**<0.001**
	Absent	142 (66)	

Bold values means statistically significant.

**Table 6:** Multivariate analysis to determine factors predicting clinical response.

Variables	P-value	Odds ratio	95% CI for Exp(B)	
			Lower	Upper
Clinical node	**0.022**	2.031	1.106	3.731
rENE	**0.030**	1.714	1.054	2.786
Site	**0.025**	1.694	1.070	2.683

Bold values means statistically significant.

## Discussion

Extranodal extension/extracapsular spread (ENE/ECS) is one of the most important independent prognostic factors in head and neck SCC (HNSCC) and has been found to show a direct impact on poor survival outcomes (12, 13). pENE has been extensively studied in the past and is considered to be the gold standard for deciding adjuvant treatment in HNSCC (12). Addition of chemotherapy to radiotherapy as adjuvant (CCRT) improves the 5-year survival rate compared to RT alone (14). In patients with LAHNSCC who do not undergo surgery, precise assessment of rENE is of critical importance for planning the appropriate treatment, for prognosticating the disease, and for predicting treatment response.

This study aimed to assess the prognostic importance of rENE detected by CT in predicting clinical response after CCRT and its implication on survival outcomes. According to the results of our study, rENE was an independent prognostic factor for DFS and showed trend toward significance for OS. A recent study by Moon et al. (7) showed similar results where rENE (+) had worse 5-year OS (74% vs. 94%, p < 0.01) and DSS (42% vs. 84%, p < 0.01) compared with the rENE (−) cohort. The other important factors affecting survival were the site of the disease, stage, and treatment arm, which are similar to the previously reported articles (2).

It is of paramount importance to accurately stage the nodal disease in patients radically treated with CTRT, which is based on clinical and radiological findings, due to its recent incorporation in the AJCC staging eight edition. It is necessary for appropriate radiation planning and intensification of treatment. Failure to do so will result in residual/recurrent disease with poorer outcomes (15, 16). We found the presence of rENE, cN, and site of primary to be an independent prognostic factor in deciding clinical response. rENE-positive patients had 1.71 times increased risk of IR than negative patients. Patients with cN had two times more chances of PR compared to node-negative patients.

In the eighth edition of the AJCC TNM classification, cENE was included for N3b stage classification and risk stratification of HNSCC because of uncertainty about the reliability of rENE (4). However, multiple studies now suggest that rENE can be reliably ascertained with high specificity if strict imaging criteria are used (17–23). Many previous reports have compared concordance between rENE and pENE (17–22). It has the potential to refine the cN classification and facilitate treatment selection in both viral-related and viral-unrelated HNSCC (24–27). Diagnosis of rENE based on our criteria has shown it to be a good prognostic factor for survival and predictor of response to treatment. The assessment of rENE with improved diagnostic accuracy is most important to reduce false-positive and false-negative results. There is a lack of consensus regarding the imaging criteria for assessing ENE from the past studies (28–31). Multiple imaging criteria were used by different studies (**Table 7**); for example, example Url et al. proposed apparent fat and soft tissue infiltration and infiltration of muscle and carotid sheath (11). A study by Aiken et al. (20) found a strong association between radiologically determined lymph node necrosis and pathological extracapsular spread (pENE) (p<0.01), a similar result showed by Randall et al. (16). A study by Carlton et al. found 48% sensitivity and 86% specificity for capsular irregularity imaging criteria (17). A recent study by Faraji et al. concluded that of the seven imaging features hypothesized to be associated with ENE-status, the presence of irregular nodal margins and absence of perinodal fat plane were the most specific and sensitive features, respectively (22).

**Table 7:** Systematic review of literature for diagnostic accuracy of imaging-based ENE versus pathological ENE as gold standard and its clinical implication.

Authors	HNSCC sub site and sample size	Number of (rENE+) and (pENE+)	Factors considered for rENE	Accuracy of rENE with gold standard as pENE [sensitivity/specificity (%)]	Inference/Clinical Application
Url et al. (11)	HNSCC (49)	(Examiner 1: 15 and Examiner 2: 16)* (17)	a) Apparent fat and soft tissue infiltration b) Infiltration of muscle, carotid sheath	Examiner 1: 73/91 and Examiner 2: 76/91	High specificity
Prabhu et al. (19)	HNSCC (432)	(46), (87)	a) Irregular borders and/or perinodal fat stranding b) Invasion of adjacent structures	23/98 30/99.7	High specificity
Aiken et al. (20)	OSCC (111)	(29), (28)	a) Irregular borders and/or perinodal fat stranding b) Invasion of adjacent structures	68/88	High specificity Central necrosis is best criteria
Maxwell et al. (21)	HPV+ OPC (65)	(19), (38)	a) Nodal capsular contour irregularity b) Poorly defined nodal margins c) Loss of intervening fat planes d) Invasion of adjacent structures	55–77/70–85	Not reliable in HPV+ cases
Carlton et al. (17)	HNSCC (93)	(Examiner 1: 32 and Examiner 2: 37) *, (56)	a) Indistinct nodal margin b) Infiltration into adjacent tissue c) Irregular nodal enhancement d) Matted nodes e) Central necrosis	Examiner 1: 57/81; Examiner 2: 66/76	Moderate specificity
Almulla et al. (10)	OSCC (483)	(55), (114)	a) Ill-defined Lymph node borders b) Matted nodes	52/96	High specificity
Noor et al. (18)	HPV + OPC (80)	(Likely ECS: 15 & 14; Definitely ECS 11 & 14)*	a) Assessing internal characteristics b) Capsule contour c) Perinodal fat stranding d) Invasion into surrounding structures	Examiner 1: 56.5/73.3; Examiner 2: 60.9/66.7	High specificity
Faraji et al. (22)	HPV+ OPC (73)	(NA), (32)	a) Indistinct capsular contours b) Irregular nodal margins c) Perinodal fat stranding d) Perinodal fat planes e) Nodal necrosis f) Intranodal cysts g) Nodal matting	Irregular nodal margins: 45/ 94 absence of perinodal fat plane: 87/ 50	Presence of irregular nodal margins and absence of perinodal fat plane were the most specific and sensitive features, respectively.
Moon et al. (7)	HNSCC (117)	(30), (NA)	Enhancement, thickening, and irregularity of nodal rim; blurred border and/or infiltration mahajan of the adjacent fat or other soft tissue planes; and infiltration of the sternocleidomastoid muscle, internal jugular vein, or carotid artery	NA	Pretreatment rENE is not only associated with CCRT response but also act as independent prognostic factor for survival in patients with HNSCC treated with CCRT.
Kang-Hosing Fan et al. (13)	HPC (355)	(171),(NA)	Infiltration of adjacent fat/muscles, irregular nodal surface, or irregular capsular enhancement	NA	rENE considered an adverse prognostic marker for survival in patients with HPC treated by primary CCRT and correlates with inferior RFS regardless of N stage.
Mahajan et al. (current study)	LAHNSCC (354)	(140), (NA)	a) Capsular irregularity with fat stranding b) Capsular irregularity with fat invasion c) Capsular irregularity with muscle/vessel invasion	NA	rENE can be reliably used as an independent prognostic marker for survival in patients with LAHNSCC.

HNSCC, head and neck squamous cell carcinoma; HPV + OPC, HPV-associated oropharyngeal carcinoma; OPC, oropharyngeal carcinoma; OSCC, oral cavity squamous cell carcinoma; HPC, hypopharyngeal cancer; pENE, pathological extranodal extension; NA, not applicable. *Two separate examiners value.

**Figure 8:**
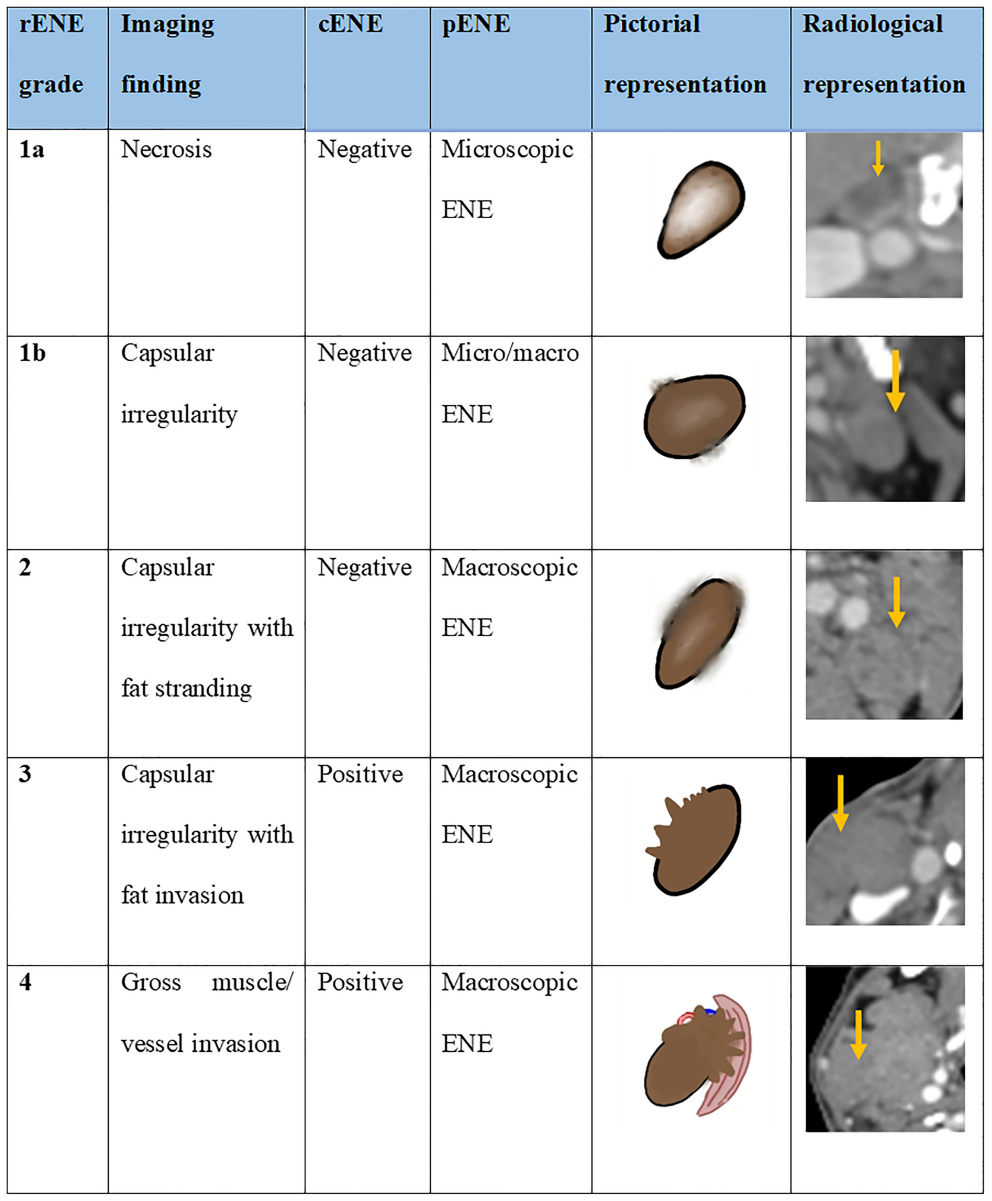
The proposed imaging-based mahajan grading system for radiological extranodal extension (rENE) and their clinical ENE (cENE) and pathological ENE (pENE) correlates.

The study by both Randall et al. and Aiken et al. found that central necrosis was the best predictor of ENE among multiple other imaging criteria. We found necrosis in 74% of rENE positive cases. Prabhu et al. found that sensitivity ofCT detection of ECS was 18% when the extent of histopathological ENE from the capsule was less than or equal to 1 mm and that sensitivity and accuracy improved as the extent increased more than 1 mm. With this in mind, we propose the imaging-based mahajan ENE grading system, which will be applicable on CT, MRI, PET-CECT, and US. The rENE grading system with its cENE and pENE correlates is shown in **Figure 8**.

There were few limitations of our study; as patients were radically treated with CCRT and only a few underwent salvage neck dissection, rENE status confirmation was not available. We also could not study the diagnosis and prognostic implication of microscopic versus macroscopic rENE. Indian population has predominance of human papilloma virus (HPV)-negative oropharyngeal cancers. Same was evident from our trial as well; of the 269 patients with oropharyngeal cancer, p16 was feasible only in 212 patients. In addition, 187 of 212 patients were p16 negative, and the analysis of the same has already been published (32). Therefore, it was not possible to study the impact of rENE in HPV-positive oropharyngeal cancers, which is another research avenue.

To conclude, pre-treatment rENE is an independent prognostic marker for survival in patients with LAHNSCC treated radically with CCRT that can be used as a potential predictive marker for response to treatment and hence stratify patients into responders vs. non-responders. We propose the mahajan rENE grading system applicable on CT, MRI, PET-CECT, and US.

## Data Availability Statement

The raw data supporting the conclusions of this article will be made available by the authors, without undue reservation.

## Ethics Statement

The studies involving human participants were reviewed and approved by IEC TMC. Written informed consent for participation was not required for this study in accordance with the national legislation and the institutional requirements.

## Author Contributions

Study concept: AM, AC. Study design: AM, AC. Data acquisition: AM, AC, VP, KP, VN. Quality control of data algorithms: AM, AC. Statistical analysis: VP, RV, AM, AC Manuscript preparation: all authors. Manuscript editing: AM, AC. Manuscript reviewing: all authors. All authors contributed to the article and approved the submitted version.

## Conflict of Interest

The authors declare that the research was conducted in the absence of any commercial or financial relationships that could be construed as a potential conflict of interest.

## Publisher’s Note

All claims expressed in this article are solely those of the authors and do not necessarily represent those of their affiliated organizations, or those of the publisher, the editors and the reviewers. Any product that may be evaluated in this article, or claim that may be made by its manufacturer, is not guaranteed or endorsed by the publisher.
